# A millennium-long climate history of erosive storms across the Tiber River Basin, Italy, from 725 to 2019 CE

**DOI:** 10.1038/s41598-021-99720-z

**Published:** 2021-10-15

**Authors:** Nazzareno Diodato, Fredrik Charpentier Ljungqvist, Gianni Bellocchi

**Affiliations:** 1 Met European Research Observatory – International Affiliates Program of the University Corporation for Atmospheric Research, Via Monte Pino snc, 82100 Benevento, Italy; 2 Department of History, Stockholm University, 106 91 Stockholm, Sweden; 3 Bolin Centre for Climate Research, Stockholm University, 106 91 Stockholm, Sweden; 4 Swedish Collegium for Advanced Study, Linneanum, Thunbergsvägen 2, 752 38 Uppsala, Sweden; 5 INRAE, VetAgro Sup, UREP, Université Clermont Auvergne, 63000 Clermont-Ferrand, France

**Keywords:** Climate sciences, Environmental sciences, Environmental social sciences, Hydrology, Natural hazards

## Abstract

Rainfall erosivity drives damaging hydrological events with significant environmental and socio-economic impacts. This study presents the world’s hitherto longest time-series of annual rainfall erosivity (725–2019 CE), one from the Tiber River Basin (TRB), a fluvial valley in central Italy in which the city of Rome is located. A historical perspective of erosive floods in the TRB is provided employing a rainfall erosivity model based on documentary data, calibrated against a sample (1923–1964) of actual measurement data. Estimates show a notable rainfall erosivity, and increasing variability, during the Little Ice Age (here, ~ 1250–1849), especially after *c*. 1495. During the sixteenth century, erosive forcing peaked at > 3500 MJ mm hm^–2^ h^–1^ yr^–1^ in 1590, with values > 2500 MJ mm hm^–2^ h^–1^ yr^–1^ in 1519 and 1566. Rainfall erosivity continued into the Current Warm Period (since ~ 1850), reaching a maximum of ~ 3000 MJ mm hm^–2^ h^–1^ yr^–1^ in the 1940s. More recently, erosive forcing has attenuated, though remains critically high (e.g., 2087 and 2008 MJ mm hm^–2^ h^–1^ yr^–1^ in 1992 and 2005, respectively). Comparison of the results with sediment production (1934–1973) confirms the model’s ability to predict geomorphological effects in the TRB, and reflects the role of North Atlantic circulation dynamics in central Italian river basins.

## Introduction

Alterations of the hydrological cycle are likely to aggravate both storm-erosivity, and other extreme rainfall-related hazards, in regions that are already particularly vulnerable to soil erosion, such as the Mediterranean countries^1^. Storms drive rainfall erosivity, overland flow (or runoff) and, in turn, erosional soil degradation, which have become one of the most prevalent major environmental issues, resulting in economic losses around the globe^2–5^. Understanding the dynamics of these damaging hydrological events, and their impacts on the landscape poses huge challenges, especially in Mediterranean environments^6^, where local-scale effects between storms and orography contrasts are evident^7^. An example of this is the river Tiber, in central Italy, at time of Roman civilization, as the naturalist Pliny the Elder (23/24 BCE–79 CE) recalls in his words (Italian translation by Castiglione, 1599, p. 50)^8^:

*Nasce il Tevere […] nell’Appennino in una montagna detta boggi la Faltona, sul Casentino, corre più di 150 miglia, riceve da quarantadue fiumi, e torrenti. Onde non è maraviglia, se quando piove dirottamente nelle montagne da tante bade ne venghi a Roma inodatione.*

The Tiber has its source in the Apennines in a mountain called boggi la Faltona, on the Casentino, runs more than 150 miles, receives from forty-two rivers and streams. So it is no wonder that when it rains heavily in the mountains from so many streams it floods Rome.

Thus, the flooding of the river Tiber was a phenomenon that the inhabitants of Rome were used to, and had to cope with, since ancient times. In particular, the flooding of the city of Rome, due to deluges (that is, floods caused by a huge amount of rainfall in a short time) and erosive storms, has been a recurring phenomenon since the times of ancient Rome, as recalled by historians Titus Livy (64/59 BCE–12/17 CE), Gaius Cornelius Tacitus (*c.* 56–*c.* 120 CE) and other writers of the Imperial Era (from 27 BCE to the fall of the Western Roman Empire in 476 CE). According to the legend about the foundation of the city of Rome, it was the river Tiber, perhaps in flood in the eighth century BCE, that dragged the basket of the legendary twin brothers Romulus and Remus, whose story tells the events that led to the founding of the city of Rome (traditionally indicated as 21 April 753 BCE) and the Roman Kingdom by Romulus (753–716 BCE). Already the Roman poet of the Augustan Era Publio Virgilio Marone (70–19 BCE), in the *Aeneid* (book 5), wrote that although the river Tiber is usually placid and peaceful in its course, making it dangerous with its floods (Carcani, 1875, p. 27)^9^:

*Sternit agros, sternit sata laeta boumque labores, Praecipitesque trahit silvas.*

It lowers the fields, destroys crops and toil, and drags the forests precipitously.

Since then, during almost every decade—with greater or lesser impetus—the waters of the Tiber have emerged from its bed, flooding large areas of the city and the surrounding countryside^10^. For instance, the Benedictine monk Paul the Deacon (*c*. 720s–796/799 CE), in his *Istoria Langobardorum*, recalled the flood of 555 CE (Frosini 1977, p. 143)^11^:

*A causa delle continue piogge il Tevere a Roma gonfiò talmente che le sue acque lambirono le mura e allagarono parecchi quartieri.*

Due to continuous rainfall, the Tiber in Rome swelled so much that its waters lapped the walls and flooded several districts.

**Figure 1:**
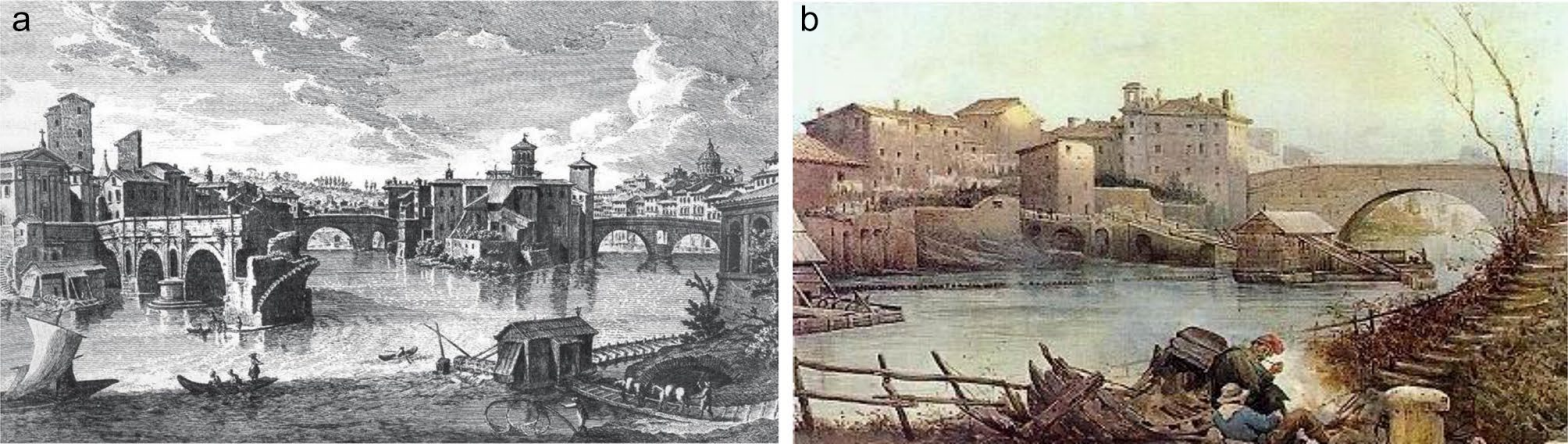
The Tiber River near the city of Rome as a source of danger as well as wealth. (**a**) Engraving by Giuseppe Vasi (1710–1782) illustrating *Ponte Senatorio oggi detto Ponte Rotto* (1748), almost completely destroyed during the flood of 1598 (https://www.nga.gov/collection/art-object-page.125672.html; image available free of charge for any use, commercial or non-commercial under Creative Commons Zero Open under Access Policy for Images of Works of Art Presumed in the Public Domain, National Gallery of Art, Washington DC, USA, https://www.nga.gov/notices/open-access-policy.html); (**b**) Painting of *Ponte Cestio ed Isola Tiberina (rione Ripa)* with the mills of SS. Annunziata and S. Francesco by Ettore Roesler Franz (1845–1907), *c.* 1880 (https://en.m.wikipedia.org/wiki/File:IsolaTiberinaEPonteCestioByRoeslerFranz.jpg; work in the public domain, content available under CC BY-SA 3.0).

However, the river was not only a danger with its floods and consequent destruction of farms, houses and bridges^12^, and mills^13^ (Fig. 1a), but at the same time an inexhaustible source of drinking water, and energy to power its historical mills (Fig. 1b).

Linking environmental and hydrological variability to human history remains constrained by the scarceness of high-resolution climatic data in medieval and earlier times^14–17^. Proxy‐based considerations of cyclone variability are restricted to evidence of long‐term changes at sparse spatial scales, limiting our understanding of the forcing imprint, and the relevance of the processes involved in cyclone intensification for historical climate periods^18^. Ljungqvist et al.^19^ furthermore identified knowledge gaps and biases in the existing scholarship on the past climate–human history nexus, including geographical biases, and a disproportionate focus on extremely cold periods, restricting our capability to produce plausible future scenarios^20^. In particular, fluctuations in climate and its extremes across different time-scales^21,22^ can produce changes in the occurrence and intensity of storms and, in turn, in the rainfall erosivity, i.e., the power of rainfall as defined by the R-factor of the Universal Soil Loss Equation (USLE) and its revised forms^23^. Rainfall erosivity is a major factor for understanding the dynamics of surface processes such as erosional soil degradation^24–26^ as well as other landscape disturbances such as floods and landslides^27^. It also offers the opportunity to capture the fingerprint of climate change, not the least in the Mediterranean region, which is a climate change “hotspot” and a particular sensitive region in terms of hydroclimatic variability^28^. An improved knowledge of the temporal variability in erosive rainfall is important because its changes indicate varying magnitudes of hydrological events that damage Mediterranean landscapes. This is of great importance for the development of conservation and environmental management plans in a changing climate^29^. The Mediterranean region is marked by a composition of gradual and abrupt changes of rainfall patterns, including variable patterns of erosive storms^30^. Deep in the warm sea, Mediterranean cyclones form mainly around a few centres, with a dominant area in the Gulf of Genoa, where slowly moving low pressure can move large amounts of precipitation^31^. Damaging hydrological events, and thus rainfall erosivity, occur here mainly due to: (1) intense and highly convective rain showers (often less than one hour in duration), with rainfall amounts generally below 100 mm that are susceptible of flash-floods, and (2) mesoscale convective rainfall causing stationary rainfall for several hours or days resulting in large rainfall and more than 200 mm in just a few hours^32^.

Already in historical times, these hydrological extremes were documented in diaries. For instance, in the third book of his work *Del Tevere* (“On the Tiber”), Andrea Bacci (a sixteenth-century Italian philosopher, physician and writer) describes a flash-flood in Rome in the year 1557 (cited from Brioschi, 1876, pp. 23–24)^33^:

*In quel dì che fu il 14 Settembre 1557, essendo tempo quasi sereno si vide in un subito ingrossare il Tevere, e da ivi a poco non senza meraviglia che pareva quasi ritornare indietro rincalzato dal mare, cominciò prima ad uscire dalle chiaviche, ed appresso dal pieno del fiume a traboccare, e scorrere sì furiosamente per tutte le strade, che in pochissime ore fece la più parte di Roma navigabile*.

On that day, September 14, 1557, when the weather was almost clear, the Tiber was seen to swell at once, and from there on, not without wonder, as it seemed almost to have been pushed back by the sea, it began first to flow out of the culverts, and then from the middle of the river to overflow, and flow so furiously through all the streets, that in a few hours it made most of Rome navigable.

Longer and large rainfalls in the year 1415 were, instead, described in the *Diarium Romanum Gentilis Delphini* and *Cod. Mss. Vatican.* (Carcani, 1875, p. 39)^9^:

*Il giorno di giovedì ultimo del mese di Ottobre dell’anno 1415 […] fu una terribile procella di venti, lampi, tuoni e pioggia: «ita quod apparebat quod totus Mundus deberet finire». Nè cessò mai di piovere, giorno e notte, fino al 25 Novembre.*

On the last Thursday of October in the year 1415 [...] there was a terrible storm of wind, lightning, thunder and rain: *« so that it was clear that the whole world ought to be to put an end ».* It never stopped raining, day and night, until 25 November.

**Figure 2:**
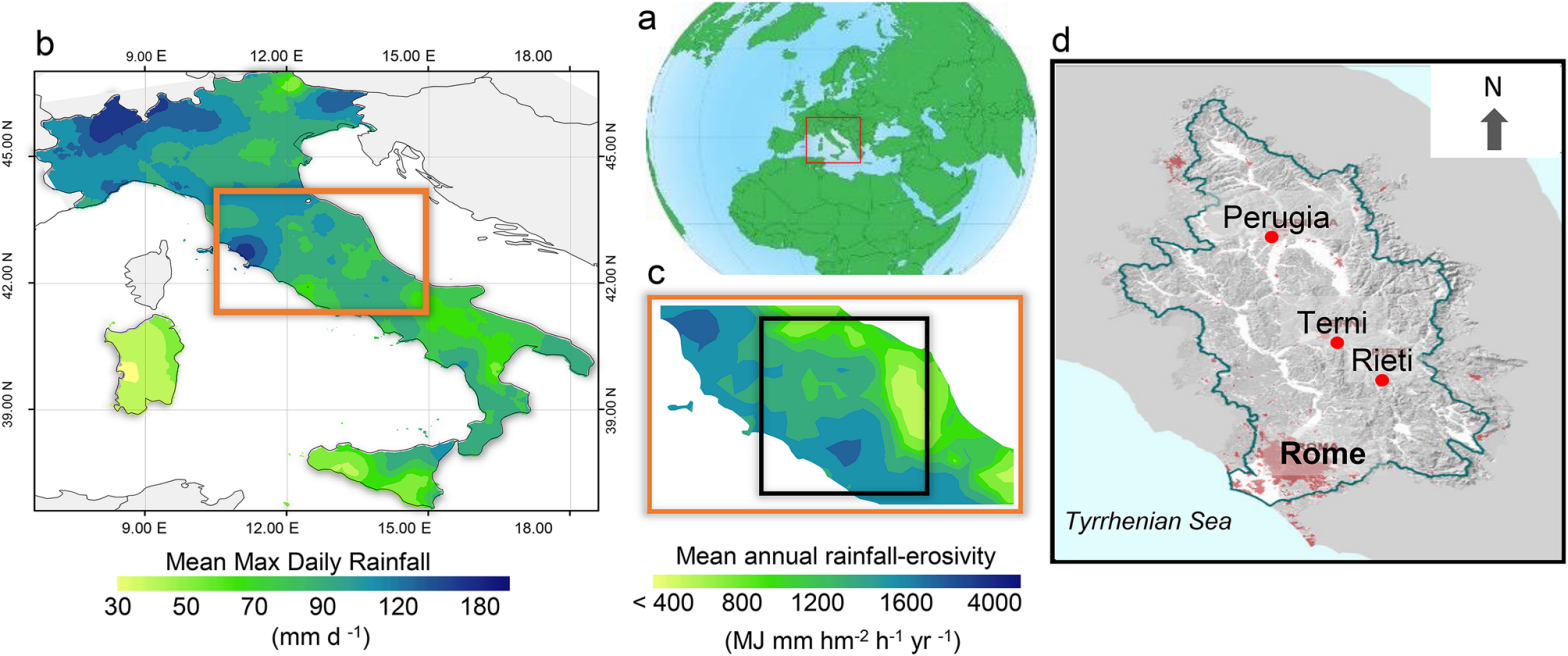
Environmental setting and rainfall patterns. (**a**) Framing of the central Mediterranean sector (red box), obtained from Collection of Free Vector Earth (http://clipart-library.com/free-vector-earth.html); (**b**) Spatial pattern of mean maximum daily rainfall (inverse distance weighting interpolation) in the period 1994–2003 (orange box indicates central Italy); (**c**) Downscaled (co-kriging interpolation) mean annual rainfall erosivity in central Italy (orange box) and across the Tiber River Basin (black box) in the 2003–2012 period (arranged from the Global Rainfall Erosivity Database—https://esdac.jrc.ec.europa.eu/themes/global-rainfall-erosivity—based on the European Soil Data Centre—https://esdac.jrc.ec.europa.eu); (**d**) Boundary of the Tiber River Basin with indication of the main cities. In (**c**), the erosivity values roughly cover the range from the third (200–400 MJ mm hm^−2^ h^−1^ yr^−1^) and the eight (3100–5200 MJ mm hm^−2^ h^−1^ yr^−1^) of 10 erosivity classes from Panagos et al.^129^. Graphs (**b**) and (**c**) are derivative maps generated using the Geostatistical Analyst extension of ArcGIS 9.1 (ESRI, S ESRI https://www.esri.com).

From these historical records, we understand that the Italian lands (Fig. 2a) have been exposed to floods and, in turn, to aggressive rainfall. This is shown by the fact that the annual maximum daily rainfall is also today likely to reach critical values in central Italy (Fig. 2b). In particular, the Tiber River Basin (TRB), the focus area of this study (Fig. 2c), is even more exposed than other Italian areas, where high erosivity produces considerable soil erosion and floods. On average (period 2003–2012), rainfall erosivity over the TRB varies from about 1000 to 3000 MJ mm hm^−2^ h^−1^ yr^−1^ (Fig. 2d, black squared). Recently published studies on the relationship between hydrological extremes and climate change^34^, including rainfall erosivity^35–37^, indicate a new focus of environmental science about the history of extreme hydroclimatic events. Indeed, there are studies regarding the relationship between erosive forcing and climate in the central Mediterranean region^38–44^, indicating renewed attention to environment–climate interactions in historical and ecological research in support of reconstruction of extreme rainfall events (including rainfall erosivity) and planning decisions, especially in agriculture. However, the Italian landscape, and that of the TRB in particular, has been shaped by stormy and erosive events (with related floods) that have influenced human life in the basin and its main city, Rome, since the Middle Ages and before. High-resolution and well-dated records are needed to understand the long-term hydroclimatic variability in this region over such a long period. In particular, rainfall records with a sub-hourly temporal resolution are required to obtain actual rainfall erosivity values compatible with the (R)USLE) methodology^23^. This makes historical studies challenging, as such detailed observations are not available before the modern instrumental period (digital measurements began systematically in the 1980s^41^).

One way to reduce the gap in rainfall detail is offered by modelling approaches, which use indirect inputs from storms and floods as extracted from historical documentary data. In this way, modelling rainfall erosivity requires exploring mechanisms inherited from past hydrological extremes like storms and floods^45^, using parsimonious models to overcome the limitations imposed by detailed models, which required detailed data and cannot be applied to historical periods^46^. In particular, low-resolution historical documentary records of extreme weather events can be adopted to help predict rainfall erosivity when satisfactory instrumental data are not available^37^. However, the floods of the Roman period, and earlier times, are almost all dated on an annual time-scale. It was not until *c*. 725 CE that monthly data became available on a more continuous basis. These data allowed us to estimate not only the intensity of flood events, but also their seasonality, and then erosivity, which cannot be predicted from annually-dated events alone. Thus, our analysis extends no further back in time than 725 CE. It is only from this period that storms and floods are sufficiently documented in historical documentary records with a monthly resolution, a prerequisite for estimating annual rainfall erosivity, which has a strong seasonal component^47^. Using weather anomalies such as storms and floods (and their variability) from historical documentary records for the TRB, the aim of this study was two-fold: (1) to develop a parsimonious model to reconstruct annual rainfall erosivity from 725 to 2019 CE, and (2) to capture a broad climate variability and, in turn, identify changes in landscape stress. Methodologically similar to other studies^48,49^ that also reconstructed rainfall erosivity time-series over past centuries, this study is unique in its length and geographical focus, exceeding those of previous studies.

## Results and discussion

### Integrity of the reconstructed extreme hydrological events

**Figure 3:**
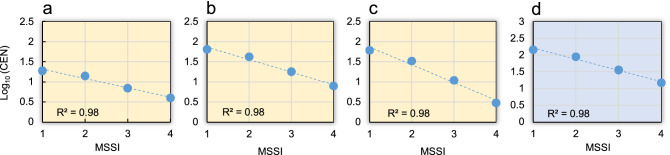
Completeness of reconstructed storms events. Cumulative distribution of the logarithm of the number of storm events versus their Monthly Storm-Severity Index (*MSSI*) in the Tiber River Basin for sub-periods (yellow-coloured graphs) (**a**) Medieval Climate Anomaly: 725–1249 CE; (**b**) Little Ice Age: 1250–1849 CE; (**c**) Current Warm Period 1850–2019 CE; and (**d**) the entire dataset: 725–2019 CE (blue coloured graph).

We identified 285 extreme hydrological events occurring in the TRB from 725 to 2019 CE. These events date back to the flooding of the Tiber River in the urban section of the city of Rome, as information attributable to the northern course of the river is only available in a fragmentary and patchy form^10^. The breakdown of these events by severity resulted in 145 *stormy* events, 89 *very stormy* events, 36 *great stormy* events and 15 *extraordinarily stormy* events (Table S1). However, our historical hydrological database is found in a variety of heterogeneous source types, including written records and effects preserved in the built environment that can help reconstruct past climate. Documents such as personal manuscripts and official records, as well as printed materials, artworks and, more recently, electronic data, pose particular problems of homogenisation^50^. It is well established, for instance, that small, localised storms can be frequent, but tend to be underestimated, especially when occurring in remote locations. To overcome some of these uncertainties in our database, we have established a reasonable standard for the events recorded (*MSSI*: Monthly Storm-Severity Index) to transform subjective information into an objective vector of data that can be investigated statistically for temporal homogeneity (see “Numerical and categorical inputs” in the section “Methods”). This was done by setting a subdivision of the time-series in three major climate sub-periods—the Medieval Climate Anomaly (MCA; here 725–1249 CE), the Little Ice Age (LIA; here 1250–1849 CE) and the Current Warm Period (CWP; here 1850–2019 CE)—and testing for each of the sub-periods (Fig. 3a–c), and for the entire dataset (Fig. 3d), the scale-invariance in the relationship between the number of events larger than storm-strength events and the events of the same strength^51^. The completeness analysis was accounted with the logarithmic relationship between the cumulative number of events (*CEN*) and the *MSSI* values in the range 1 ≤ *MSSI* ≤ 4, as follows:

1 $${log}_{10}\left({CEN}_{i,j}\right)=a+b\cdot {MSSI}_{i,j} \,\mathrm{with}\, i=1,\dots , 4\, and\, j=1,\dots ,3$$

where *i* and *j* indicate severity class and sub-period, respectively. In total, 285 events were extracted in the range 1 ≤ *MSSI* ≤ 4, which are represented in qualitative terms as *stormy*, *very stormy*, *great stormy*, and *extraordinary stormy*. The negative slopes in the three sub-periods, and in the entire dataset, reflect a downward trend in frequency as storms become more severe. With coefficients of determination *R*^*2*^ = 0.98, it can be assumed that the storms in the 725–2019 CE period are scale-invariant.

### Rainfall erosivity model assessment

**Figure 4:**
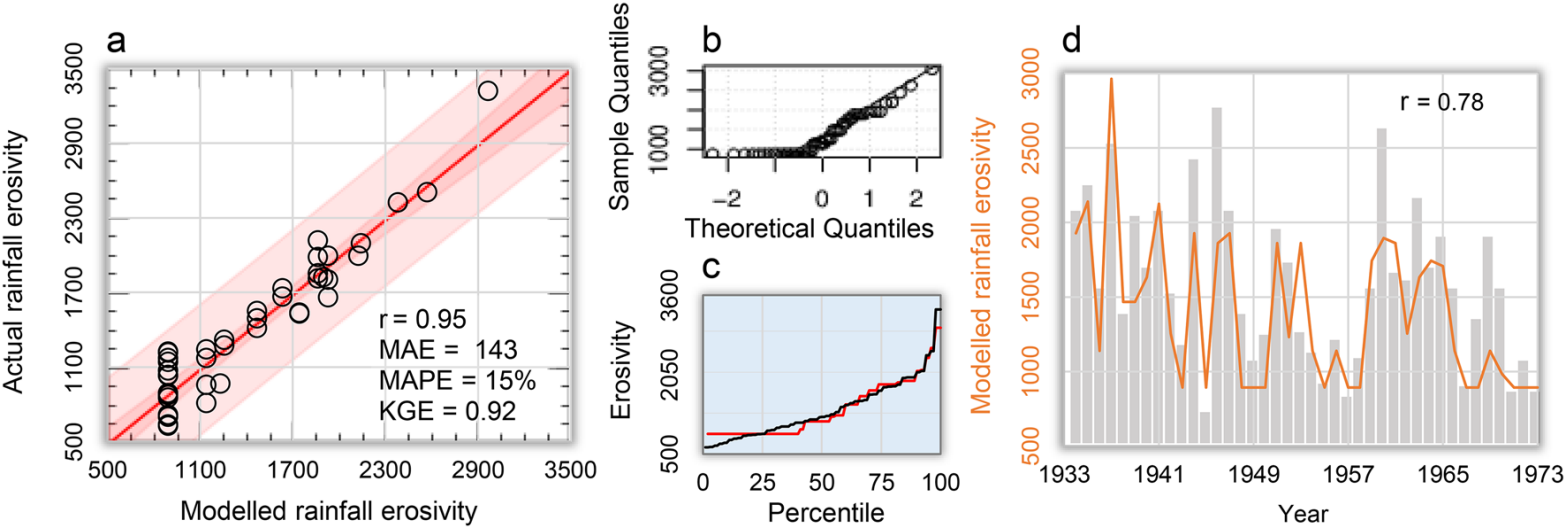
Rainfall erosivity model calibration and indirect validation for the Tiber River Basin. (**a**) Scatter-plot (dotted black regression line and red line of identity) of actual versus modelled—Eq. (5)—rainfall erosivity (MJ mm hm^−2^ h^−1^ yr^−1^) for the 1923–1964 period, with the inner bounds showing 90% confidence limits (power pink coloured area), and the outer bounds showing 95% prediction limits for new observations (light pink); the MAE in the box in a) is also in MJ mm hm^−2^ h^−1^ yr^−1^; (**b**) Q-Q plot (theoretical versus sample quantile values); (**c**) Percentiles of actual (blue curve) and modelled (orange curve) rainfall erosivity; (**d**) Co-evolution of modelled annual rainfall erosivity (orange curve) and suspended sediment yield (grey histogram) at the mouth of the river Tiber in the 1934–1973 period^130^.

Using our simplified, non-linear multivariate additive model (*REM*_*TRB*_), we estimated annual values of mean areal rainfall erosivity over the TRB (*REM*_*TRB*_) taking into account (via *MSSI* inputs) the interrelationship between spatial patterns of hydroclimate and storm erosivity, consistent with a sample (1923–1964) of detailed (R)USLE-based data obtained for the study area—Eq. (2)—after removing the 1965 outlier of 2875 MJ mm hm^−2^ h^−1^ yr^−1^ (~ 2% of the dataset of 43 years), not considered for model calibration (not shown in Fig. 4a). We obtained an annual mean areal erosivity value of 1357 MJ mm hm^−2^ h^−1^ yr^−1^ (± 537 MJ mm hm^−2^ h^−1^ yr^−1^ standard deviation) over the study period, which is close to the mean of actual data of 1360 MJ mm hm^−2^ h^−1^ yr^−1^ (± 568 MJ mm hm^−2^ h^−1^ yr^−1^ standard deviation). The actual values varied between 631 (in 1945) and 3322 (in 1937) MJ mm hm^−2^ h^−1^ yr^−1^, while the calibrated model gave 893 (in several years) and 2964 (in 1937) MJ mm hm^−2^ h^−1^ yr^−1^ as minimum and maximum estimates, respectively. The calibrated parameter values of Eq. (5) are: A = 73.83 MJ mm^−1^ hm^−2^ h^−1^ yr^−1^, B = 1.00 MJ mm^−1^ hm^−2^ h^−1^ yr^−1^, α = 6.00, β = 3.00, γ = 2.05 and k = 2.00. Other parameters were calibrated for Eq. (2)—$\Phi$=3.2 –, Eq. (3)—φ = 0.100, Ω = 0.696, ν = 0.5—and Eq. (4)—ψ = 0.3.

With these values, the linear regression between actual and estimated erosivity data is statistically significant (F-test *p* ~ 0.00), with intercept a = –1.214 (± 70.595 standard error) MJ mm hm^−2^ h^−1^ yr^−1^ and slope b = 1.003 (± 0.048 standard error). The R^2^ statistic (goodness of fit in the scatter-plot of Fig. 4a) indicates that the *REM*_*TRB*_ explains 95% of the variability. MAE (mean absolute error) equal to 143 MJ mm hm^−2^ h^−1^ yr^−1^ is lower than the standard error of the estimates (180 MJ mm hm^−2^ h^−1^ yr^−1^). MAPE (mean absolute percent error) equal to 12% and Kling-Gupta Efficiency (KGE) equal to 0.92 also indicate satisfactory model performance and efficiency. The Durbin-Watson (DW) statistic close to 2 (DW = 2.57) implies that there is no significant (*p* = 0.98) lag-1 autocorrelation (*k*) in the residuals (*k* = –0.30). Figure 4b indicates the normal approximation of quantiles pattern (normality test *p* = 0.48^52^; test for equal distributions of actual and estimated data *p* = 0.10^53^). The distribution shapes of the modelled (red curve) and observed (black curve) erosivity data indicate a satisfactory prediction (Fig. 4c), especially after the 40th percentile, in an area of the distributions that includes the median and the highest values. An indirect validation (performed against a dependent variable on the one assessed) was also obtained by comparing the modelled rainfall erosivity with the sedimentation processes that occurred in the TRB during in the period 1934–1973. Figure 4d shows that the predicted erosivity (orange curve) can be an important driver of sediment transport processes (grey histogram) occurring in the TRB. This independent validation (over a period of time different from the calibration period) indicates that the model output is highly correlated with the suspended sediment delivered through the TRB (*r* = 0.78), which shows an overall downward trend, albeit with some large fluctuations. Satisfactory agreement with sediment production in the basin was also obtained in 1965 (and subsequent years), the year whose observed value was not considered for model calibration. Thus, although anomalous situations such as that of 1965 may occur from time to time (and be found in the calibration dataset), they are not likely to change the structural characteristics of the erosivity model and its ability to interpret geomorphic processes in the basin.

### Historical reconstruction of rainfall erosivity

**Figure 5:**
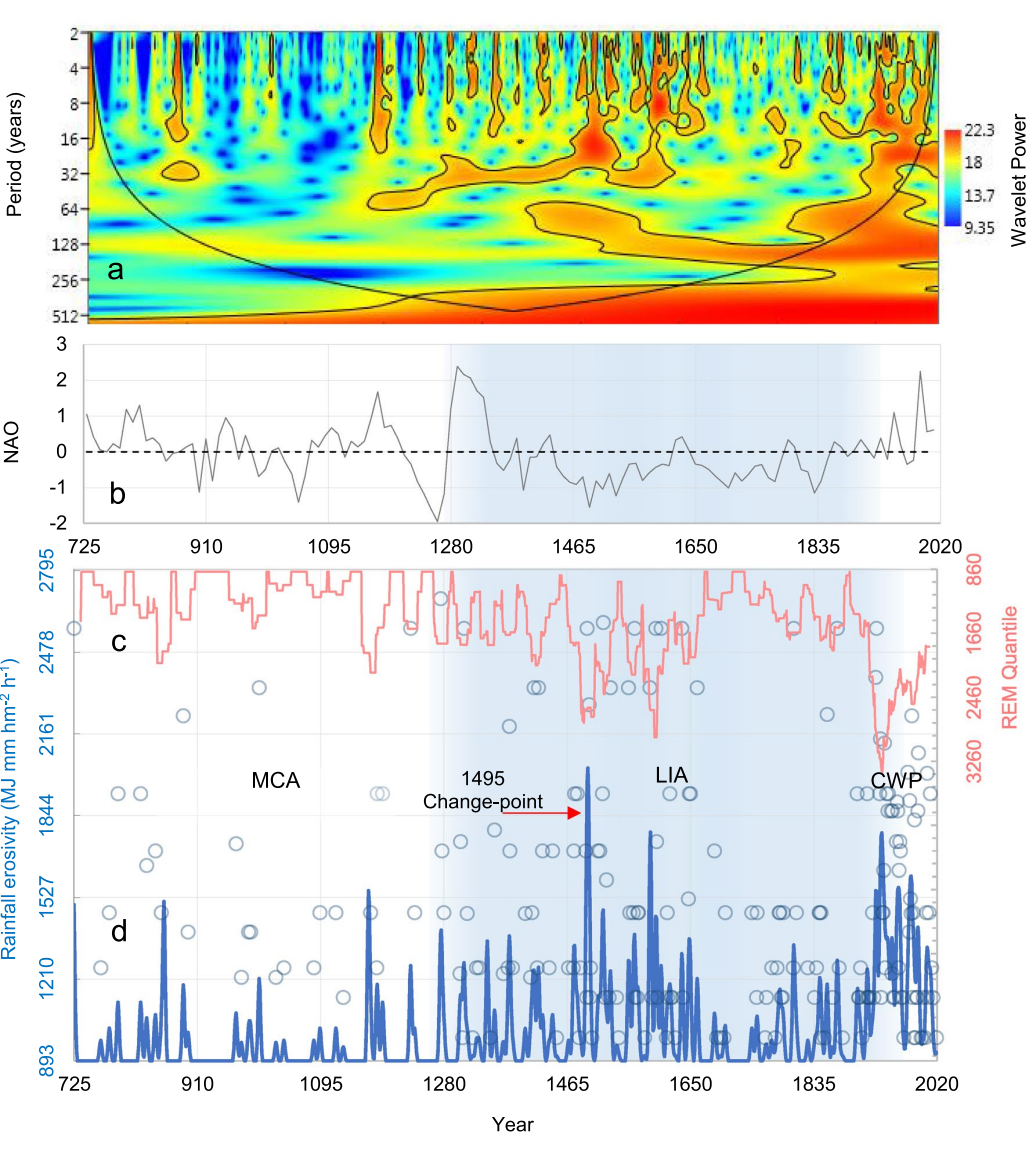
Rainfall erosivity and climatic patterns over the 725–2019 CE period in the Tiber River Basin. (**a**) Wavelet spectrum of the rainfall erosivity time-series with bounded colours identifying 0.05 significance areas (the bell-shaped, black contour marks the limit between the reliable region and the region below the contour where the edge effects occur, a.k.a. cone of influence (**b**) North Atlantic Oscillation (annual NAO from Hernández et al.^100^); (**c)** Trend of quantile-rainfall erosivity with return period T = 50 years (red curve); (**d)** Evolution (red arrow indicating the change-point year 1495) of estimated annual rainfall erosivity (blue circles) with the relative smoothed trend by the 11-year Gaussian function (bold blue curve). The *y*-right axes in (**c**) is in the reverse-scale. MCA: Medieval Climate Anomaly (725–1249 CE); LIA: Little Ice Age (1250–1849 CE); CWP: Current Warm Period (1850–2019 CE). The LIA is identified as a light blue area across the graphs (**b**–**d**).

Figure 5 shows the erosive forcing and other features that have occurred over the historical course on the landscape of the Tiber River Basin. In particular, Fig. 5d shows the evolution of the areal-mean annual erosivity values (blue circles) during the 725–2019 CE period, as obtained by Eq. (5). The long-term areal-mean value of estimated erosivity data is 1005 (± 335 standard deviation) MJ mm hm^−2^ h^−1^. To detect possible trends and oscillations in the discrete values, and to compare contemporary and historical patterns, the time-series of annual rainfall erosivity was smoothed with the filtered 11-year Gaussian function (Fig. 5d, bold blue line). The pattern of quantiles, with 50-year return period, was also shown (Fig. 5c, red curve). From the smoothed trend it can be seen that from the LIA onwards, erosivity is characterized by two major oscillations, within which there is a marked inter-annual variability.

This contrasts with the MCA, during which the estimated erosivity fluctuates more erratically. Before the year 1000, the MCA is characterized by relatively moderate erosivity (on average 941 MJ mm ha^−1^ h^−1^ yr^−1^), rarely (in 12 years) exceeding 1200 MJ mm hm^−2^ h^−1^ yr^−1^, in a period when Italy and the Tiber River Basin experienced intense deforestation and colonisation activity^54^, favoured by religious orders, especially the Benedictines^55,56^. After the year 1000, the mouth of the river Tiber shows intermittent activity (no longer suitable for navigation since 1118), attesting to frequently reduced river flows^57^.

Longman^58^ found a similar distribution for the Eastern Mediterranean region, where erosivity reflects less rainy conditions during the MCA, with relatively low hydroclimatic variability. With the onset of the LIA, however, erosivity became more vivid, with notable values often exceeding 1400 MJ mm hm^−2^ h^−1^ yr^−1^ (in 11 years between 1250 and 1400). Stormy events had also affected other regions at that time, with disastrous consequences in much of Western Europe^59^.

Entering the hydrologically most active central part of the LIA, change-points in the erosivity time-series were detected well before the Maunder Minimum of reduced solar activity (*c*. 1645–1715 CE^60^): in 1474 with the Buishand^61^ (1982) statistic, and in 1495 and 1497 with the Standard Normal Homogeneity Test-double shift^62^. These change-points mark a transition towards a more extreme rainfall erosivity in a period (late fifteenth century) characterised by afforestation, with the reforestation of grasslands^56^, or sometimes the joint expansion of grasslands (meadows and pastures) and forests^63^. In particular, the flood of the river Tiber in the year 1495 (red arrow in Fig. 5d) has been handed down to us by direct testimonies of its disruptive force, and among them the most interesting is that of the Venetian oratories (ambassadors) who wrote^64^:

*Dopo due giorni e mezzo di quel turbine di pioggia il 4 dicembre il cielo torno sereno. Tosto il Tevere cominciò a gonfiare con straordinaria celerità allagando tutta la citta bassa […] Le acque toccavano la sella dei nostri cavalli. Ad un'ora di notte la piena giunse anche alla nostra via.*

After two and a half days of this whirlwind of rain, the sky cleared up again on 4 December. Soon the Tiber began to swell with extraordinary speed, flooding the entire lower city […] The waters touched the saddle of our horses. At one hour of the night the flood reached even our street.

As the fifteenth century progressed, floods became increasingly frequent. In the two centuries between 1400 and 1600 CE, floodplain forest returned to the valley and mesic forest expanded on the slopes^65^. The change-point in the year 1495 (2570 MJ mm hm^−2^ h^−1^ yr^−1^) marks a crossroad after which nothing will resemble the previous centuries, with erosivity becoming more aggressive and changeable, with erosive storms tending to oscillate more, as the smoothed values also show. Thus, the sixteenth century was undoubtedly the most hydrologically damaging for the TRB, with five of the most disastrous floods that Rome has ever known, in the years 1530, 1557, 1589 (with 2340 MJ mm hm^−2^ h^−1^ yr^−1^), 1590 and 1598 (with 3531 and 2570 MJ mm hm^−2^ h^−1^ yr^−1^, respectively). In 1530, as Rome was beginning to recover from the sack carried out in 1527 by the German mercenaries of Charles V (1500–1558), Holy Roman Emperor from 1519 to 1556, the Tiber River continued the work of destruction begun three years earlier.

In fact, going into the details of the chronicles of the time, it is possible to identify the characteristics of each of them. Such, for instance, is the report given in the more succinct but explanatory chronicle by Ludovico Gomez (bishop of Sarno, died in 1543) who wrote *De prodigiosis Tiberis inundationibus ab Urbe condita ad annum M.D.XXI*, printed in Rome in 1531 (Frosini, 1977, p. 160)^11^:

*Era già sul levar del sole il sabato mattina, dell'8 del mese di ottobre, quando il Tevere mossosi fuor del solito letto, comincio a versare montagne d'acqua*.

It was just before dawn on Saturday morning, 8 October that the Tiber emerged from its usual banks and began pouring down mountains of water.

However, the most ruinous of all was that of December 1598, and from its description we can see that it affected an immense area of the city of Rome (D’Onofrio, 1980, p. 160)^66^:

*Da che si conosce che i diluvij antichi sono stati maggiori di questo [1598] che avanzò però quello del 1557 e quello del 1530 piu alti di quanti ne erano segnati ne marmi che si trovano incastrati ne' muri di case e Chiese e altri luoghi per Roma, chio ho potuto ritrovare.*

It is known that the ancient floods were larger than this one [1598], but the one of 1557 and the one of 1530 were higher than those marked in the marbles that are set in the walls of houses and churches and other places in Rome, which I could find.

To conclude, the sixteenth century was a period of concentrated storms with different spatio-temporal scales: we have in fact a mean erosivity of 1087 (± 494 standard deviation) MJ mm hm^−2^ h^−1^ yr^−1^, while the extreme values are 3531 (in 1590), 2593 (in 1519), and 2570 (in 1566) MJ mm hm^−2^ h^−1^ yr^−1^, corresponding to return periods of > 100 anni for the first value and about 50 years for the second and third. Count Onofrio Castelli (1580–*c.* 1658) claims that indiscriminate deforestation was the cause of the great floods of the Tiber that occurred at that time (Brioschi, 1876, p. 28)^33^:

*Mentre nei monti, sono selve e boschi, gli alberi, sterpi, erbe e cose simili, ritengono qualche parte dell’acqua, e qualche parte ne è succhiata dalla terra, e nelle selve per lo più il suolo è disuguale, e fa seni e concavità, le quali pure l’acqua ritengono. Il rimedio dunque sarebbe il non permettere il continuarsi dell’estirpare le selve e i boschi, ma concedere solo il legname, senza svellere le radici.*

While in the mountains, which are forests and woods, the trees, brushwood, grasses and the like retain some of the water, and some of it is sucked up by the earth, and in the forests the soil is mostly uneven, and makes sinuses and concavities, which also retain the water. The remedy, therefore, would be not to allow the forests and woods to continue to be uprooted, but only to allow the wood to be harvested, without uprooting the roots.

With a mean rainfall erosivity of 1006 MJ mm hm^−2^ h^−1^ yr^−1^ (± 338 MJ mm hm^−2^ h^−1^ yr^−1^ standard deviation), similar to that of the previous century, the seventeenth century was though less aggressive, with storm erosivity exceeding the value of 1700 MJ mm hm^−2^ h^−1^ yr^−1^, corresponding to a return period of about 20 years, only in the years 1606, 1619, 1637, 1648, 1650, 1660 and 1686. From the mapping of the watch-towers near the river mouth^67^ it can be deduced that, after the great floods mentioned above, the main mouth advanced rapidly towards the sea. The eighteenth century saw a remarkable decline of annual erosivity (on average 957 MJ mm hm^−2^ h^−1^ yr^−1^) and its variability (± 100 MJ mm hm^−2^ h^−1^ yr^−1^ standard deviation), only rarely reaching 1400 MJ mm hm^−2^ h^−1^ yr^−1^ (estimated value of 1468 mm hm^−2^ h^−1^ yr^−1^ in 1702, 1742, 1750, 1783, 1784 and 1789). A resumption of storm-erosivity activity took place in the nineteenth century (on average 985 MJ mm hm^−2^ h^−1^ yr^−1^, with the estimated peak of 2570 MJ mm hm^−2^ h^−1^ yr^−1^ in 1805 and 1870), when deforestation increased again in the upper Tiber River Basin (Brighenti, 1860, p. 9)^68^:

*Il progressivo, e crescente disboscamento per cui salirono tanto in alto i prezzi del legname […] Anche l’aspetto delle colline e dei monti mutato di selvoso in coltivato […] Nelle pioggie dirotte le acque accumulate non trovano impedimento dagli alberi.*

The progressive and increasing deforestation, which caused timber prices to rise so high […] The appearance of the hills and mountains also changed from wild to cultivated [...] In heavy rain, the accumulated water is not impeded by the trees.

**Figure 6:**
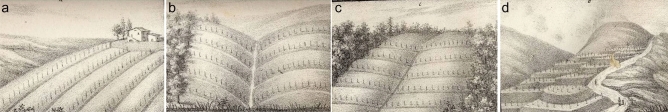
Tillage adopted in agriculture to contain soil erosion at the beginning of the nineteenth century in the Tiber River Basin: (**a**) slope with upright arrangement (*rittochino*) (**b**) hillock-cutter arrangement (*cavalcapoggio*) (**c**) riding on horseback (*tagliapoggio*) and (**d**) terracing arrangement (*ciglioni*) (arranged from ref.^70^).

In fact, forest cover in the basin area fell from 69% in 1800 to 62% in 1900 (based on the HYDE3.2 historical land-use database, https://www.pbl.nl/en/image/links/hyde). Today we can imagine how damaging these storms were for the farmers due to the erosive action of the rains by looking at the annals^69^ of Italian agriculture^70^, which illustrate the different processes—those that perhaps best preserve the material traces of the age-old work of constructing the agrarian landscape (in conflict with climatic and anthropogenic forces)—to which the steep terrain was subjected in order to mitigate the loss of soil that was carried downstream (Fig. 6).

By the end of the nineteenth century, Sacheri^71^ (1901) recalls the famous flooding of the Tiber River in December 1870: an extraordinary and imposing flood that inundated the city of Rome and the surrounding countryside, and on the 28th and 29th reached 17.22 m above the zero level of the Ripetta hydrometer (in a river wharf located along the upper-most part of the urban course of the Tiber River), a height that contemporaries could hardly remember as being comparable with the greatest floods that had occurred several centuries earlier.

During the twentieth century (1248 MJ mm hm^−2^ h^−1^ yr^−1^ on average ± 456 MJ mm hm^−2^ h^−1^ yr^−1^ standard deviation), erosive forcing resumed, exceeding 2000 MJ mm hm^−2^ h^−1^ yr^−1^ in 1928 (2380), 1929 (2570), 1935 (2141), 1937 (2964), 1976 (2013), 1982 (2231), 1992 (2087) and 2005 (2008), and remaining critically high thereafter (e.g. 1928 MJ mm hm^−2^ h^−1^ yr^−1^ in 2010). These values are similar to those of the sixteenth century, but with longer return periods (about 100 years for the first two extremes: 2964 and 2570 MJ mm hm^−2^ h^−1^ yr^−1^ in 1937 and 1929). These annual rates are remarkable, considering that they are smoothed over the basin area, which means that even much larger erosive events may have occurred locally in the basin. From 1980 onwards, however, an overall decrease in both annual rainfall erosivity and its extremes is observed. According to Sharma^72^, basin-wide erosivity would decrease due to the smaller storm extent at this scale: with fewer major storms there is less major flooding and less erosivity, especially extreme erosivity. This is consistent with the projected decrease in mean winter storm precipitation and dynamic weakening of cyclones over the Mediterranean region^73^.

However, the attribution of extreme erosive events associated with floods and heavy rains to climate change signals remains uncertain, not the least due to the high spatial variability and long-term unpredictability of these events^74^. As aggressive precipitation becomes more frequent with global warming^75^, climate hazards may already have become more changeable and unpredictable on a small scale in disaster-affected areas of the central Mediterranean^76^. Gentilucci^77^ also showed a trend towards more extreme rainfall events in central Italy during the 1961–2017 period. However, this occurs with different patterns of change over small areas with no consistent spatial and temporal trends emerging^78^. So, even if Raible with co-authors^79^ support a purely thermodynamically driven increase in cyclone-related precipitation extremes, the way Mediterranean cyclones have produced trends in rainfall extremes in recent decades remains difficult to understand^80^, with significant negative trends of cyclone frequency in spring often offset by positive trends in summer^81^.

Summary statistics for three climatic sub-periods (Table 1) show that rainfall erosivity has continued to increase on average from medieval times to the recent warming period (from 941 to 1159 MJ mm hm^−2^ h^−1^ yr^−1^), with the highest coefficients of variation (35–36%) and percentiles (e.g. 98th percentile around 2300 MJ mm hm^−2^ h^−1^ yr^−1^) during the LIA and CWP. However, although the completeness graphs provide an overall view of the reliability of the dataset over different sub-periods (Fig. 3), a more disaggregated view (44 in the MCA, 133 in the LIA and 108 in the CWP) shows that the early medieval period may be characterised by a scarcity of documentary sources and thus of accounts of hydrological disasters. This makes the interpretation of the *MSSI* more difficult and there may be underestimates of rainfall erosivity for that period, as we do not know how much of this underestimation is due to lack of information and how much to the actual non-existence of the phenomenon. Some underestimation may however be associated with events of lesser impact, as high-magnitude hydrological events (e.g. with *MSSI* = 3 or *MSSI* = 4) are more likely to be remembered in memory and historical records, as opposed to low-magnitude events (e.g. with *MSSI* = 1 or *MSSI* = 2) that may sporadically affect an area and thus not be recorded. This calls for an update of the dataset and model estimates as new documents come to light.

**Table 1:** Descriptive statistics for three climatic sub-periods of the modelled time-series of rainfall erosivity.

Climatic sub-period	Mean (MJ mm hm^−2^ h^−1^ yr^−1^)	Coefficient of variation (%)	Percentiles (MJ mm hm^−2^ h^−1^ yr^−1^)	
			95th	98th
MCA (725–1249 CE)	941	27	1217	1721
LIA (1250–1849 CE)	1017	35	1707	2345
CWP (1850–2019 CE)	1159	36	2011	2325

MCA: Medieval Climate Anomaly; LIA: Little Ice Age; CWP: Current Warm Period.

### Influence of solar and teleconnection cycles

The wavelet power spectrum (Fig. 5a) reveals significant high-frequency periodicities in the erosivity time-series with a scattered ~ 11-year cycle, together with a more regular ~ 22-year period during the LIA as well as during the recent warming phase. Both periodicities are key features of solar activity variability, with the ~ 22-year magnetic solar cycle composed of two ~ 11-year sunspot cycles with opposite polarities^82,83^. While significant periodicities less than ∼11 years occasionally occur without any relation with climatic periods, other low-frequency periodicities are also significant. The quasi-secular periodicity appearing during the late fifteenth century to the present reflects the periodicity of ~ 100-year cycle of Gleissberg^84^, while the periodicity of > 300 years extending over the central part of the LIA (and beyond but outside the reliable region formed by the time axis and the bell-shaped contour) may reflect combinations of the ~ 210-year Suess/de Vries cycle and the Gleissberg cycle^85,86^.

The Sun can affect the hydrological cycle through feedbacks at multiple scales, which can lead to complex geographical distributions of solar‐related signals in hydroclimatic factors. Within a river basin, in particular, the integrated nature and inertia of sediment discharges can reveal pulses driven by the Sun that amplify precipitation signals, while the links between solar activity and precipitation can be weak^87,88^. Here, reconstructed floods (translated into rainfall erosivity data) can be considered as suitable proxies for the Tiber River discharges. Zanchettin et al.^89^ provided Sun-like periodicities related to sunspot magnetic activity as an indication of solar influence on river flood discharges, and suggested that the Sun could be one of the precursors of hydrological processes in northern Italy.

Diodato et al.^48^ also found statistical links between precipitation hazard metrics (erosivity density and return periods of maximum erosivity values) and the ~ 22-year solar cycle and Atlantic teleconnection patterns in northwestern Italy over the 1701–2019 CE period. These studies provide evidence that regional patterns of precipitation or temperature changes can be modified by large‐scale climate teleconnections induced by changes in the absorption of solar energy in the atmosphere and ocean^90^. In Europe, the strength of solar activity apparently modulates the connection between the frequency of regional precipitation oscillation peaks and the persistence of oceanic and atmospheric patterns over the North Atlantic region^91,92^. We refer here to circulation patterns, which are reflected by the Atlantic Multidecadal Variability (AMV) or its internally generated component, commonly referred to as the Atlantic Multidecadal Oscillation (AMO), i.e., the variability of the sea-surface temperature over a timescale of several decades^93^. As also reported for the Arno River Basin (1000–2019 CE) in central Italy^49^, we observe that higher rainfall erosivity values in the TRB tend to be associated with dominant warm phases of the AMV (reconstruction by Wang et al.^94^ from 800 to 2010 CE, not shown). Sea-surface temperature anomalies also induce atmospheric pressure gradients^95^, redistributing air masses between subtropical and subpolar latitudes of the North Atlantic, and modulating the strength and latitudinal location of westerly flows^96,97^.

The North Atlantic Oscillation (NAO), in particular, measures the fluctuations in the difference of air pressure at sea level between the Icelandic Low (at ~ 38°N) and the Azores High (at ~ 65°N). Major influences of the NAO on precipitation regimes in the central Mediterranean are documented^98,99^ and our analysis support a relationship between rainfall erosivity in the TRB and the proxy-based multi-annual NAO reconstruction (Fig. 5a) of Hernández et al.^100^. In particular, we observe that the intensification of erosive forcing after the end of the fifteenth century corresponds to the transition to substantially negative NAO values in the fifteenth century (-0.28 on average, ± 0.09 standard error, Student-t *p* ~ 0.00), which follows a long phase (725–1475 CE) with no clear dominance of positive or negative NAO states (0.12 on average, ± 0.10 standard error, Student-t *p* = 0.21).

However, the NAO index presents large inter-seasonal and interannual variability, particularly the winter NAO, which also has substantial decadal and longer time-scale variability^101^. The negative phase of the NAO dominated the Atlantic circulation between the mid-1950s and the 1970s, followed by an abrupt transition to positive phases during the winter of 1979–1980 (with the atmosphere persisting in this mode during the winter of 1994–1995) and a sharp slowdown^102^. Furthermore, the high spatio-temporal variability of rainfall regimes due to topography and the presence of sea masses make it difficult to establish the actual role played by the NAO on erosive precipitation in southern Europe^103^, especially when wide areas are considered and long-term time-series (i.e. over a millennium) are not available^43^. We thus highlight the need to understand in more detail the links between large-scale atmospheric and oceanic variability and rainfall erosivity, which depend on the geographical context (e.g. large regions or catchments) and the time scale (e.g. annual or decadal) over which the hydrological response is assessed. To this end, we advocate the use of a limited-area (basin-scale) model on time scales longer than a millennium because, as this study shows, it can be appropriate to assess changes in hydrological responses that are controlled by large-scale circulation patterns. The most intense and localized precipitation events are, however, subtle and difficult to detect when working with annual-scale time-series.

We envision that in the future will be able to conduct an in-depth analysis with reconstructed series that will enable to better highlight the most likely trend in a changing climate. With this in mind we conclude this article with a quotation from Brioschi (1876, p. 17)^33^:

*L’antica Roma che ebbe tanto a soffrire del Tevere, non ci ha nulla lasciato che potesse mettere un freno per sempre alle inondazioni, noi abbiamo da lei a prender alcun esempio la cui ricordanza ci metta nella via di più vaste ricerche*

Ancient Rome, which suffered so much from the Tiber, has left us nothing to stop the floods forever; we can take from it an example whose memory will put us on the road to a wider search.

## Methods

### Study area and climate

**Figure 7:**
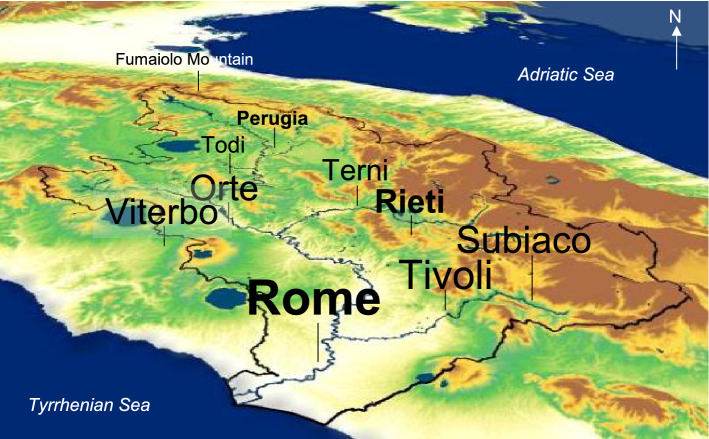
Perspective view of Tiber River Basin (watershed limits in black) from the North (Mount Fumaiolo, 43°47′N, 12°04′E). The Tiber River (in blue) winds through the landscape, passing near the city of Perugia (43°06′N, 12°23′E), after leaving its source, then near Todi (42°46′N, 12°24′E), and flow towards the mouth through the city of Rome (41°53′N, 12°30′E). Its tributary Aniene flows westward past Subiaco (41°56 ′N, 13°06′E) and Tivoli (41°57′N, 12°47′E). The map with the cities and geographical names overlaid is an output image created by the authors from Arm of Carabineers website (http://www.carabinieri.it/editoria/natura/la-rivista/home/tematiche/ambiente/progetti-sul-tevere).

Formerly called Albula (after the Latin *albus* = white referring to the light colour of its blond waters), or Thybris (from the Etruscans) or Rumon (linked to the ancient Etruscan-Latin name of Rome), the Tiber is the main river of central and peninsular Italy. With 405 km of course, it is the third longest Italian river (after the Po, 652 km, and the Adige, 410 km). The source of the Tiber river is on the slopes of Mount Fumaiolo (1268 m a.s.l.), on the side turning towards Perugia (upper Tiber River Basin, Fig. 7), near Balze, a village of Verghereto (in the province of Forlì-Cesena). The Tiber River Basin is rich in tributaries and sub-tributaries, but the river receives most of its water from the left bank, where its main adductors are the Chiascio-Topino system, the Nera and the Aniene. The tributaries of the right bank are the Nestore with Caina and Fersinone, the Paglia (with the Chiani) and the Treja, between the provinces of Rome and Viterbo. The Tiber River also passes in the vicinity of Perugia, Marsciano, Deruta and Todi, while Tivoli and Subiaco are crossed by the Aniene tributary, at east (Fig. 7).

The river was used for many centuries as a means of communication: in Roman times merchant shipping could go directly to Rome, to the Emporium at the foot of the Aventine, while smaller vessels suitable for river navigation transported goods and agricultural products from Umbria, through a capillary navigation system that penetrated the region also through the tributaries, in particular Chiascio and Topino.

From a climatic point of view, the TRB can be divided into three sub-regions (Fig. 7): the flat sub-region, which includes the area surrounding the city of Rome and the rural areas northeast of the city (areas coloured in white tending towards yellow and green); the sub-region of the Apennine valley along the main branch of the Tiber, which also includes the surrounding mountainous territories, such as Todi and Perugia (areas coloured in green); and the third sub-region at east, mainly mountainous, which includes the provinces of Terni and Rieti (areas coloured in ocher brown). The climate of the first sub-region is temperate, with a hot summer and an annual rainfall of 800–1000 mm. The second and third sub-regions are the most interesting from a hydrological point of view, as they are the main source of deluges and floods, which play an important role in driving the most important erosive storms of the year. The climate is mainly continental, with moderately hot summers in the valley bottoms (roughly province of Rieti), and not very hot summers in the hills (roughly province of Perugia). The amount of annual rainfall is about 800 mm at Perugia, and over 1000 mm at Rieti. The rainfall is distributed over 80–100 days per year, with two peaks: the main peak in autumn and the second in late spring. Summer and autumn storms are common in all three sub-regions.

### (R)USLE-compliant actual rainfall erosivity data

To develop and calibrate a parsimonious model for rainfall erosivity estimation in the study-area, we used the areal-mean of actual rainfall erosivity (MJ mm hm^−2^ h^−1^ yr^−1^) for the TRB (*R*_TRB_), as calculated for each year of the 1923–1965 period, by adapting an equation originally developed by Diodato^41^:

2 $${R}_{TRB}= \Phi \cdot \sqrt{{Pa}_{TRB}\cdot {dx}_{TRB}\cdot {hx}_{TRB}}$$

where Φ is a scale parameter to convert the term under square root into the rainfall erosivity unit (MJ mm^−1^ hm^−2^ h^−1^ yr^−1^), *Pa*_TRB_ is the areal-mean annual precipitation (mm yr^−1^) as derived from GPCC (Global Precipitation Climatology Centre) 0.25° × 0.25° Full Data Monthly Product Version 2020^104^ via Climate Explorer (http://climexp.knmi.nl), while *dx*_TRB_ and *hx*_TRB_ are the areal-mean maximum annual daily (*dx*, mm d^−1^) and hourly (*hx*, mm h^−1^) rainfall, respectively, estimated annually as:

3 $${dx}_{TRB}=\left(\varphi +{MSSIi\left(max\right)}_{i\in Y}\right)\cdot \sqrt{{dx}_{RO}}+{\Omega \cdot dx}_{TRB(NCEP)}$$

4 $${hx}_{TRB}=\psi \cdot \left[1-0.4\cdot cos\left(6.28\cdot \frac{m-1.5}{23-m}\right)\right]\cdot {dx}_{TRB}$$

where *MSSI* is the Monthly Storm-Severity Index for which the maximum value is taken from the group of months *Y* (*i* = 1, 2, 3, 4, 8, 9, 10, 11, 12) as in Eq. (5), *m* = 1, …, 12 is the month of year in which *dx*_*TRB*_ takes place, *dx*_RO_ is the annual daily maximum rainfall (mm d^−1^) from the Roman College Observatory^105^ and *dx*_TRB(NCEP)_ is the annual daily maximum rainfall (mm h^−1^) from the NCEP–Reanalysis data^106^ via Climate Explorer. The two equations include empirical parameters of position ($\varphi$) and scale ($\Phi$, $\Omega$, $\psi$).

**Figure 8:**
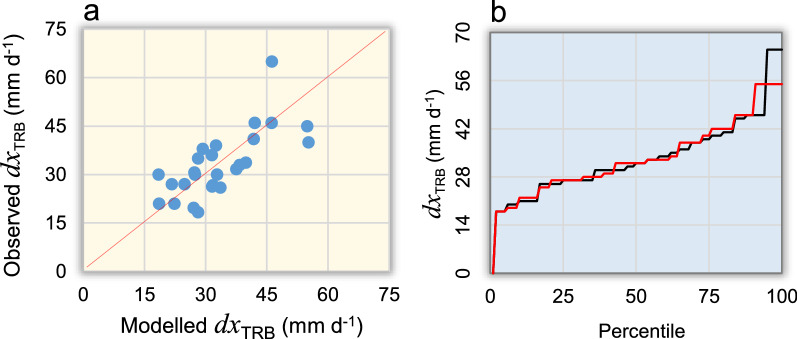
Observed and modelled annual maximum daily rainfall (*dx*_TRB_): (**A**) Scatter-plot between observed and modelled data, (**B**) the related percentile distributions.

The main issue was to obtain a homogeneous and continuous areal series of *dx* and the only way was to find a correlation between the years in which the basin was covered by a sufficient number of recording rain-gauges so as to have a homogeneous observed series of *dx*_*TRB*_, and a corresponding number of homogeneous and continuous predictors, *dx*_*RO*_, ${MSSIi\left(max\right)}_{i\in Y}$ and *dx*_*TRB(NCEP)*_ that could give us a sufficiently strong relationship to estimate rainfall erosivity over the *dx* basin area for the entire 1923–1965 period. The scatter-plot in Fig. 8 shows a sufficient proximity between the modelled *dx*_*TRB*_ data, Eq. (3), and the observed *dx*_TRB_ data available from the former SIMN (*Servizio Idrografico e Mareografico Nazionale*) and now present in the *Annali Idrologici* project (https://www.isprambiente.gov.it/it/progetti/cartella-progetti-in-corso/acque-interne-e-marino-costiere-1/progetto-annali). In particular, we obtained annual areal values of *dx*_*TRB*_ by averaging the data observed by weather stations in the basin in the years when data from at least 30 recording rain-gauges were available. In this way, we aimed to cover more than 70% of the basin area based on high-quality rainfall data.

The high-magnitude/low-recurrence erosivity value of 2875 MJ mm hm^−2^ h^−1^ yr^−1^, occurred in 1965 was excluded from the *R*_*TRB*_ actual series because it gives a strong indication of an anomalous value, likely due to the occurrence of a notable rainfall event in the basin, concentrated on September 3. Similar erosivity values obtained in other years tended to be closely associated with pronounced precipitation and non-zero *MSSI* values in different months, whereas in 1965 *MSSI* = 3 occurred in September while it was equal to zero in each of the other months. The rain that fell on September 3 proved to be erosive but did not produce sufficient force to develop a flood outside September (which in fact was not recorded in the chronicles of the time). Given the rarity of such situations, their representation is somewhat hampered by the low resolution of the model, Eq. (5), which does not attempt to capture the details of rainfall erosivity fluctuations.

### REM—Rainfall erosivity model

For the historical estimation of annual rainfall erosivity (MJ mm hm^−2^ h^−1^ yr^−1^), we performed a regression model, hereafter referred to as the Rainfall Erosivity Model for the Tiber River Basin (*REM*_*TRB*_), which uses the *MSSI* data and their variability as inputs. The non-linear derivation of rainfall erosivity from rainfall intensity was obtained with a parsimonious approach comparable to the scenario depicted by (R)USLE-based erosivity data^23^. The non-linear model of annual erosivity in the TRB takes the following form:

5 $${REM}_{\mathrm{TRB}}=\mathrm{A}\cdot \left[\mathrm{\alpha }+{\left(\sum_{\mathrm{i}\in \mathrm{X}}{\mathrm{MSSI}}_{\mathrm{i}}\right)}^{\mathrm{k}}\right]+\mathrm{B}\cdot \left[\upbeta +{\mathrm{SD}}_{\mathrm{i}\in \mathrm{Y}}{\left({\mathrm{MSSI}}_{\mathrm{i}}\right)}^{\mathrm{k}}\right]\cdot \left(\upgamma +\sum_{\mathrm{i}\in \mathrm{Z}}{\mathrm{MSSI}}_{\mathrm{i}}\right)$$

where: *A* (MJ mm^−1^ hm^−2^ h^−1^ yr^−1^) and *B* (MJ mm^−1^ hm^−2^ h^−1^ yr^−1^) are scale parameters converting the output of two dimensionless terms of the model into the result unit; α, β and γ are shift parameters predicting rainfall erosivity when the monthly input values (*i* = 1, …, 12 months) of the storm-severity index (*MSSI*_*i*_) are all equal to zero; *k* is a shape parameter; SD is standard deviation; X (*i* = 8, 9, 10, 11), Y (*i* = 1, 2, 3, 4, 8, 9, 10, 11, 12) and Z (*i* = 1, 2, 3, 4, 12) are different groups of months (the symbol $\in$ stands for “belongs to”).

**Figure 9:**
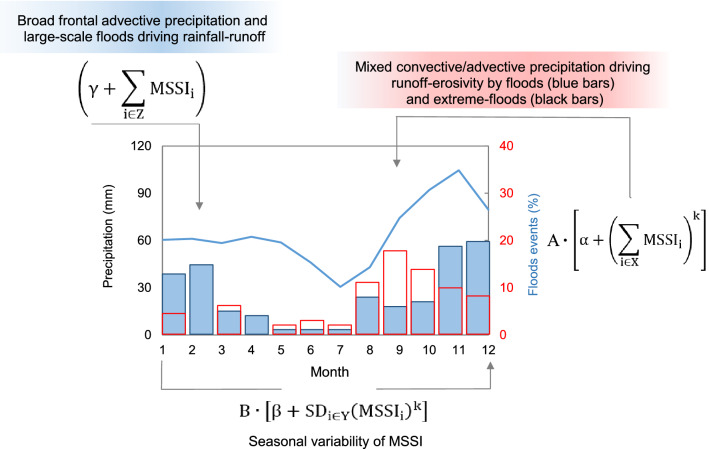
Monthly precipitation and frequency of floods and extreme floods (1940–2016) over the Tiber River Basin. Areal monthly rainfall totals (blue line) are from GPCC (Global Precipitation Climatology Centre) 0.25° by 0.25° monthly land-surface precipitation dataset^104^. Percentages of monthly floods (blue bars) and extreme floods (red-contoured bars) are from this study and ref.^32^, respectively. The terms of Eq. (5) are reported.

The idea of the *REM*_*TRB*_-model is summarised in Fig. 9. Erosive precipitation is dominated by complex climatic features, which depend on rainfall-runoff and flood generation mechanisms, and it is difficult to separate the climatic component from natural variability. According to Waldam^107^, the non-linear relationships that are created in river basins depend on the processes that dominate given hydrological regimes. Monthly rainfall, for instance, is not sufficient to describe these non-linear processes (Fig. 9, blue curve). In the central Mediterranean, erosive storms are expected to occur mainly in autumn (Fig. 9, red-contoured bars), when storm episodes are closely associated with convective processes that drive flash-floods^30,108–110^. In late autumn and winter, on the other hand, precipitation (of long duration and low intensity), generally caused by extensive frontal activity, transports large volumes of rainwater (through stratiform orographic precipitation) leading to large-scale hydrological processes such as flooding^111^. Late spring and summer rains are likely to cause a different regime of hydrological extremes than August–October, when extreme flooding is more frequent, meaning that August–April is a key time window in the year to estimate the rainfall erosivity of fluvial basins.

The separation between convective and advective events is important because convective precipitation (which is short and intense) is more dynamic and variable than advective precipitation events, which tend to be more static (Fig. 9, shaded blue box). The seasonal gap in rainfall characteristics is governed by convective processes, which are more frequent and dynamic in late summer (when rain showers dominate), or by a variable mixture of convective and advective precipitations, which are more frequent in autumn (when overland flows dominate, Fig. 9, light red shaded box). These processes are mostly captured in the REM_TRB_—Eq. (5)—by the storm-severity index (*MSSI*). Convective events, or those of mixed convective-advective nature (whose high kinetic energy causes local showers and leads to splash-erosivity) are interpreted by the standard deviation (SD) term.

### Trend assessment

The quantile approach was applied to identify step trends at any *REM*_*X*_ with return period T = 50 years. The T for the annual rainfall erosivity falling above the *j*th quantile was ranked using the lognormal distribution, according to Aronica and Ferro^112^:

6 $${Q\left({REM}_{Xt-w}\right)}_{T}=\mathrm{exp}\left[\upmu \left({REM}_{Xt-w}^{*}\right)+{u}_{T}\cdot \sigma \left({REM}_{Xt-w}^{*}\right)\right]$$

where ${Q\left({REM}_{Xt-w}\right)}_{T}$ is the *j*th rainfall erosivity density (*REM*_*X*_) quantile of the log-normal distribution with assigned return period; $\upmu \left({REM}_{Xt-w}^{*}\right)$ and $\sigma \left({REM}_{Xt-w}^{*}\right)$ are the mean and the standard deviation of the variable *REM*_X t_* = ln(*REM*_*X t*_). The subscript *t*-*w* indicates the calculation of a generic variable at time *t* over a 22-year moving window (*w*); *u*_*T*_ is the log-normal dimensionless coefficient of 2.05 for *T* = 50 years^44^. The 22-year moving window has proved effective in showing long-term trends, smoothing out secular variation and more volatile changes from one year to the next^113^.

### Model calibration and assessment

To evaluate the model statistically and graphically, analyses were carried out with STATGRAPHICS (http://www.duke.edu/~rnau/sgwin5.pdf), WESSA (https://www.wessa.net) and AgriMetSoft (https://agrimetsoft.com) online calculators. For the calibration of the parameters in Eq. (5), the first condition was to minimize the mean absolute error (optimal, 0 ≤ MAE < ∞, MJ mm hm^−2^ h^−1^ yr^−1^). The second condition was to maximise the coefficient of determination (0 ≤ R^2^ ≤ 1, optimal), which is the variance explained by the model. As a third condition, we approximated the unit slope of the regression line of the actual versus modelled data (b = 1, optimal).

Other performance indices were also calculated. The mean absolute percent error (MAPE) offers the advantage of being scale-independent and intuitive (e.g. the model is reasonable with MAPE < 30% and very accurate with MAPE < 10%). The Kling-Gupta index (− ∞ < KGE ≤ 1) was used as a measure of efficiency, with KGE > –0.41 indicating that the model is a better predictor than the mean of observations^114^. A second efficiency metric, the Nash–Sutcliffe index (− ∞ < EF ≤ 1, optimal^115^) was also calculated to assess model performance uncertainty, as EF > 0.6 indicates narrow parameter uncertainty^116^. To select the set of inputs important for the parsimonious modelling of the actual erosivity, we iteratively added predictors, one at a time until satisfactory solutions with small MAE and large R^2^ values were obtained. A third criterion was added for the final selection, i.e. |b − 1|= min. Each predictor was repositioned for > 50 iterations until convergence. The Durbin-Watson statistic was calculated to test whether the residuals were auto-correlated. ANOVA p-values were used to present the statistical significance of the regression between actual data and estimates. The wavelet power spectrum with Morlet basis function was presented as a time–frequency plot to identify potential nonstationary oscillations at different frequencies in the time-series, using the Paleontological Statistics Software Package for Education and Data Analysis (PAST^117^).

### Numerical and categorical inputs

In a first phase, research was carried out in the major archival and library centres in Italy. Then, the documentary sources were consulted by means of a web search (https://books.google.com), which generated ~ 100,000 bibliographical records. In this way, we collected a massive number of bibliographic sources but only ~ 300 records have met the criterion of including the keywords *abundant rainfall*, *storm*, *downpour*, *diluvial*, *flood* and *alluvial* (*piogge abbondanti*, *tempesta*, *diluvio*, *nubifragio*, *piena*, *inondazione*, *alluvione*), as well as some Latin locutions (e.g. *magnae pluviae, aqua maxima, diluvium, excrescentia fluminum, inundatio*) that were chosen for careful reading. Useful information was found, however, only in the following documentary sources: Castiglione^8^, Bacci^118^; Bonini^119^; Chiesa and Gambarini^12^; Carcani^9^; Brioschi^33^; Gregorovius^120^; Betocchi^121^) and in others recent studies^122–126^. For the most recent Tiber River floods we refer to the Autorità di Bacino Distrettuale dell’Appennino Centrale (http://www.autoritadistrettoac.it/notizie/l-opera-maxima) and Perugia Meteo (http://www.perugiameteo.it/home/Che-tempo-ha-fatto/Eventi-meteo-di-rilievo1/Episodi-piovosi-intensi-collegati-alle-piene-del-fiume-Tevere-1976---2007.aspx). In this way, data series were extracted by converting the information included in the historical accounts into numerical values on an index scale (see Methodological criteria and Catalogue of Monthly Storm-Severity Index (*MSSI*) in Methods and Supplementary Information). The transformation process required a dynamic understanding of the historical information used in the analysis, with a thorough knowledge of regional and sub-regional climates, and familiarity with the relative strengths and weaknesses of each type of source. A procedure, called *weather hindcasting*^127^, was used to become familiar with well-documented anomalies in the instrumental period before analysing similar cases in the pre-instrumental epoch. Storms occurring during the early part of the summer season (May to July) were not considered in this study, as they generally only affect small or isolated areas of the TRB and are not representative for storm reconstruction. A scoring system^128^ was then established to classify the *MSSIs* in the period of the year between August and April. The *MSSIs* were graded as: *0-normal*, or average storm or storm that went unnoticed, with no comment on its severity or its impacts on society and the economy: *1-stormy*, or heavy rainfall with limited damage, with isolated flooding recorded; *2-very stormy*, heavy rainfall with some flooding occurred; *3-great stormy*, or extreme rainfall event, with severe and extensive flooding, agricultural works suspended and urban communications suspended; and *4-extraordinarily stormy*, or sporadic, very extreme event, with a century-long recurrence rate (these extreme flood events affect several river basins at the same time, killing people and animals, and knocking down trees).

This kind of understanding is exemplified in the form of a table (Table S1), which incorporates monthly and annual values, and their sources for exemplary years. The study was based on a systematic and critical analysis of the data concerning the above-mentioned phenomena provided by the Italian documentary sources. For most of the information it was possible to carry out an *event check* considering more than one documentary source on the same event. It was also possible to contextualise storms with other types of historical events (e.g. social, agricultural, and religious). In this way, the reliability of information was assessed by going beyond quantitative data and explore other sources of information such as diaries, newspapers, chronicles and local stories.

## Supplementary Information

Supplementary Information.

## Data Availability

All data used in this study are freely available. Spatial patterns of mean annual rainfall erosivity over the European region (Fig. 2) are freely available from the ESDAC (European Soil Data Centre) dataset at https://esdac.jrc.ec.europa.eu/content/global-rainfall-erosivity. The proxy-based NAO dataset (Fig. 5) is available at https://doi.pangaea.de/10.1594/PANGAEA.921916. Areal mean of annual precipitation for the Tiber River Basin datasets were retrieved from the Global Precipitation Climatology Centre (GPCC) through http://climexp.knmi.nl. The full set of raw data and the equations that support the findings of this study (erosivity model, time-series reconstruction, precipitation data and storm-severity index inputs), are available in the Supplementary Table S1.
